# Impact of general anaesthesia on endoplasmic reticulum stress: propofol *vs.* isoflurane

**DOI:** 10.7150/ijms.36265

**Published:** 2019-09-07

**Authors:** Eun-Hye Seo, Liyun Piao, Hyun-Jun Park, Ji Yeon Lee, Mijung Sa, Chung-Sik Oh, Seung-Hyun Lee, Seong-Hyop Kim

**Affiliations:** 1 BK21 Plus, Department of Cellular and Molecular Medicine, Konkuk University School of Medicine, Seoul, Korea.; 2 Department of Infection and Immunology, Konkuk University School of Medicine, Seoul, Korea.; 3 Department of Anesthesiology and Pain medicine, Konkuk University Medical Center, Konkuk University School of Medicine, Seoul, Korea.; 4 Department of Microbiology, Konkuk University School of Medicine, Seoul, Korea.; 5 Department of Medicine, Institute of Biomedical Science and Technology, Konkuk University School of Medicine, Seoul, Korea.

**Keywords:** Propofol, Isoflurane, Endoplasmic reticulum stress, Reactive oxygen species

## Abstract

**Background**: This study investigated the effects of propofol and isoflurane on endoplasmic reticulum (ER) stress in an animal model under general anaesthesia.

**Methods**: Rats were randomly divided into Propofol and Isoflurane groups. Anaesthesia was maintained with propofol for Propofol group or isoflurane for Isoflurane group during 3 h. ER stress from lymphocytes in blood and tissues was evaluated between two groups after euthanasia. Reactive oxygen species (ROS) from lymphocytes in blood and tissues, and cytokines in blood were also checked. An immunohistochemical assay for ER stress marker from tissues was performed.

**Results**: After anaesthesia, the levels of CCAAT-enhancer-binding protein homologous proteins (CHOP) in blood and liver were significantly higher in Isoflurane group, compared to Propofol group [blood, 31,499 ± 4,934 (30,733, 26,441-38,807) mean fluorescence intensity (MFI) in Isoflurane group* vs.* 20,595 ± 1,838 (20,780, 18,866-22,232) MFI in Propofol group, *p* = 0.002; liver, 28,342 ± 5,535 (29,421, 23,388-32,756) MFI in Isoflurane group *vs.* 20,004 ± 2,155 (19,244, 18,197-22,191) MFI in Propofol group, *p* = 0.020]. ROS in blood was significantly higher in Isoflurane group, compared to Propofol group. However, cytokines in blood and immunohistochemical assays in tissues were similar between groups.

**Conclusion**: Significant higher of ER stress from blood and liver were observed in rats under anaesthesia with isoflurane, compared to those that received propofol. ROS from blood also showed significant higher under anaesthesia with isoflurane. However, these findings were not associated with any changes in cytokines in blood or immunohistochemical assay in tissues.

## Introduction

Anaesthesia produces a stress response, although it also alleviates noxious stimuli during surgery. The induction and maintenance of anaesthesia occur due to the actions of intravenous and/or inhalation anaesthetic agents. Intravenous anaesthetic agents are generally known to have anti-inflammatory properties 1-3. By contrast, inhalation anaesthetic agents have protective properties with preconditioning effect at ischemia-reperfusion injury 4,5. However, the differences between intravenous and inhalation anaesthetic agents do not have any significant clinical implications 6-8.

The endoplasmic reticulum (ER) is an organelle in eukaryotes that involves cell homeostasis including transport of folded proteins. Only properly folded proteins can be transported from the ER to the Golgi apparatus. An increase in the number of unfolded proteins leads to ER stress under harmful conditions 9. Studies on the influence of anaesthetic agents on ER stress under specific diseases have been conducted but no study has reported on the association between an anaesthetic agent and ER stress 10,11.

We hypothesised that an intravenous anaesthetic agent with its anti-inflammatory properties would show less ER stress, compared to an inhalation anaesthetic agent. To test this, we investigated the effects of propofol and isoflurane on ER stress in an animal model undergoing general anaesthesia.

## Materials and Methods

### Experimental design

This study was conducted after approval of the Konkuk University Institutional Animal Care and Use Committee (KU17056). Male Sprague-Dawley rats (age 6-8 weeks; body weight 200 g) were purchased from Orient Bio (Seongnam, Korea). The animal experiments were carried out based on the National Institutes of Health guidelines for care, and all animals were handled for 7 days before starting the experiments. Anaesthesia was induced by an intraperitoneal injection of 20 µg/g xylazine (Rompun^®^, Bayer Korea, Seoul, Korea). Anaesthesia was detected by pinching the hind foot. Tracheal intubation was performed on a surgical platform. A heating pad was placed between the surgery platforms to maintain warmth during surgery. After the rats were placed in the supine position, they were fastened to the platform with tape, and their tongues were pulled out with forceps. All rats were intubated using a 16 gauge 4.5 cm catheter (BD, Franklin Park, NJ, USA) inserted through the larynx to the bronchus. The correct position of the catheter for intubation was confirmed by checking for symmetrical chest expansion. A ventilator (Harvard Apparatus, Holliston, MA, USA) was connected to the catheter. The ventilator settings were as follows: fraction of inspired oxygen 0.5, inspiratory flow rate of 170 mL/min, tidal volume of 1.2 mL, and respiratory rate of 80 breaths/min 12. ER stress was checked using blood from the tail vein to confirm the baseline status at the induction of anaesthesia. The rats were randomly allocated to a Propofol group and an Isoflurane group before anaesthesia by opening a sequentially numbered envelope containing the randomization assignment. The allocation sequence was generated by Konkuk University Institutional Animal Care and Use Committee that was not otherwise involved in the trial, with random-permuted block randomization. Anaesthesia was maintained with propofol or isoflurane, according to the groups. The propofol (10 mg/mL; Dongkook Pharmaceutical, Seoul, Korea) was contained in a 50 mL syringe and continuously administered using an infusion pump (Masterflex L/S peristaltic pump with Masterflex L/S easy load pump head and L/S tubing, Cole-Parmer Instrument Co., Vernon Hills, IL, USA) at 5 µL/g/h (50 µg/g/h) via the tail vein. Isoflurane (3 vol%; JW Pharmaceutical, Seoul Korea) was administered using a vaporizer via the catheter. Anaesthesia was maintained until the rat was sacrificed by cervical dislocation 3 h after inducing anaesthesia. ER stress from lymphocytes in the blood, the liver and the kidneys was evaluated between the two groups after euthanasia. Reactive oxygen species (ROS) in the blood, the liver, and the kidneys, as well as cytokines in the blood were also checked. An immunohistochemical assay for ER stress markers was performed in the brain, the liver and the kidneys.

### Isolation of cells from the brain, the liver, the kidneys and the blood

After the rats were euthanized, the skull was cut from anterior to posterior along the dorsal midline, and the brain was separated and fixed in 4% paraformaldehyde for immunohistochemical assay. The abdomen was dissected, and the liver and the kidneys were isolated. Blood samples were obtained from an abdominal artery puncture with heparin-coated 2 mL syringes and collected into tubes precoated with ethylenediaminetetraacetic acid (EDTA). The liver and the kidneys tissues were divided into two parts. One was fixed in 4% paraformaldehyde for immunohistochemical assay and the other was minced into 1 mm^3^ pieces on ice. After the minced tissues were washed, the tissues were digested with 1 mg/mL collagenase type 1 (Sigma-Aldrich, St. Louis, MO, USA) in 5 mL phosphate-buffered saline (PBS) at 37°C for 60 min. After incubation, the cells in the digested solution were filtered through a 70 µm cell strainer (SPL Life Science, Seoul, Korea). Lymphocytes were isolated from the blood, the liver and the kidneys, to assess ER stress and to detect ROS, using density-gradient centrifugation over a Biocoll gradient solution (Biochrom, Berlin, Germany).

### Flow cytometry to assess ER stress from lymphocytes in the blood, the liver and the kidneys

Flow cytometry was performed to assess the level of ER stress. Lymphocytes from the blood, the liver and the kidneys were stained with CCAAT-enhancer-binding protein homologous protein (CHOP, DDIT3/CHOP Antibody; LS Bio Sciences, USA). The staining was performed for 30 min in the dark at room temperature, and the samples were analysed on a flow cytometer. The data were analysed using FlowJo software (Tree Star, Hillsboro, OR, USA).

### Flow cytometry to detect ROS from lymphocytes in the blood, the liver, and the kidneys

After washing lymphocytes, they were stained with 2,'7'-dichlorofluorescein diacetate (H2DCFDA; Life Technologies, Carlsbad, CA, USA) to detect intracellular ROS. The staining was performed for 30 min in the dark at room temperature, and samples were analysed on a flow cytometer. The data were analysed using FlowJo software.

### Cytokines in the blood

Serum levels of interleukin (IL)-2, interferon (IFN)-γ, tumour necrosis factor (TNF)-α, and transforming growth factor (TGF)-β were checked using enzyme-linked immunosorbent assays.

### Immunohistochemical assay for ER stress marker in tissues from the brain, the liver and the kidneys

Immunohistochemical staining for CHOP was performed to detect ER stress marker in the tissues form the brain, the liver and the kidneys. The fixed tissues were embedded in paraffin and transverse paraffin sections 4 μm thick were mounted on saline-coated slides. The mounted sections were washed in 0.01 M PBS containing 0.3% Triton X-100 (pH 7.4, PBS-T) and then immersed in 2% normal horse serum in PBS for 2 h at 37°C. They were incubated overnight at 4°C with CHOP antibody in PBS containing 1% bovine serum albumin. The sections were washed three times in PBS for 5 min and incubated in biotinylated horse-anti-mouse immunoglobulin G (1:200, Boster Biotechnology, Shanghai, China) in PBS for 2 h at room temperature. The sections were washed three times in PBS for 5 min and incubated in avidin-biotin-peroxidase complex solution (ABC, 1-100, Boster Biotechnology) for 2 h at room temperature. They were rinsed three times again in PBS for 5 min. After the staining procedure, the sections were counterstained with haematoxylin. Then the sections were dehydrated through an ethanol and a xylene series before coverslips were added with Permount^®^. Samples were visualised by incubating tissues for 2 min in 0.04% 3, 3-diaminobenzidine (DAB, Sigma) containing 0.01% H_2_O_2_. Rat immunoglobulin G (1:200, Biomeda Corp., Foster City, CA, USA) was used instead of primary antibody as a negative control. The number of CHOP-positive cells in the same area were counted and examined for optical density using ImageJ software (National Institutes of Health, Bethesda, MD, USA).

### Western blotting for ER stress from lymphocytes in the blood

Western blotting was performed to confirm the intensity of CHOP staining. Proteins were extracted from homogenised tissue samples (50 μg/lane), electrophoresed on a Tris-glycine gel (Invitrogen, Carlsbad, CA, USA), and transferred to a polyvinylidene difluoride membrane. Immunoblotting was performed by incubating the membrane in 5% fat-free dried milk overnight at 4°C, followed by incubation with anti-CHOP antibody (1:300, overnight at 4°C; Santa Cruz Biotechnology, Santa Cruz, CA, USA). After washing three times in Tris-PBS, the membranes were incubated for 1 h at room temperature with a peroxidase-conjugated secondary antibody (1:50,000). The membranes were developed using enhanced chemiluminescence (Amersham Pharmacia Biotech, Parsippany, NJ, USA). Equivalent protein loading of each lane was confirmed by stripping and re-blotting each membrane for glyceraldehyde 3-phosphate dehydrogenase (1:2,000 for 30 min at room temperature, secondary 1:20,000 for 30 min at room temperature; Biodesign International, Saco, ME, USA). The analyses were repeated in triplicate to ensure reproducibility. Band intensity was quantified using a Luminescent Image Analyzer LAS-3000 (FUJIFILM Medical Systems, Tokyo, Japan). The density of each band was analysed using ImageJ software.

### Statistical analyses

There has been no report for the association between an anaesthetic agent and ER stress to determine sample size with power analysis. Therefore, sample size was calculated using the resource equation method, instead of power analysis. With the formula for the resource equation method (E = Total number of animals - Total number of groups, any sample size, which keeps E between 10 and 20, should be considered to be adequate.), total number of animals between 12 and 22 was adequate for sample size determination 13,14. Analyses were performed using the IBM SPSS Statistics 21.0 software package (IBM Corp., Armonk, NY, USA) and GraphPad Prism 6.0 software (GraphPad Software, La Jolla, CA, USA). Differences between the two groups were detected by the *t*-test. All data are expressed as the number of rat or mean ± standard deviation (median, interquartile range). A *p*-value < 0.05 was considered significant.

## Results

Twelve rats were enrolled in the study and evenly allocated into the two groups without any drop-out from the study.

**Figure 1:**
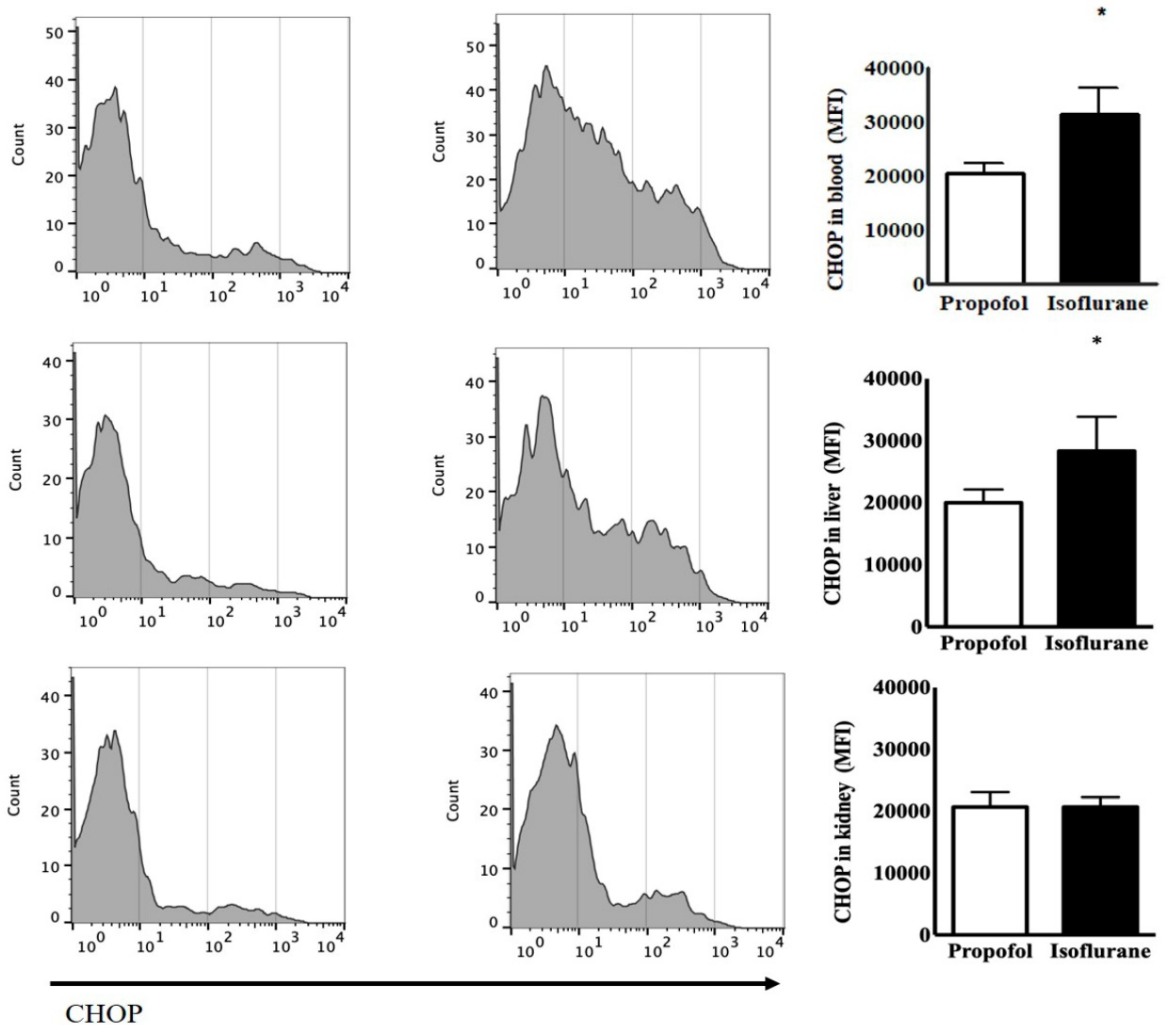
Endoplasmic reticulum stress from lymphocytes in the blood, the liver and the kidneys before and after anaesthesia with propofol and isoflurane. **Abbreviations:** CHOP, CCAAT-enhancer-binding protein homologous protein; MFI, mean fluorescence intensity. ^*^p < 0.05. CHOP from lymphocytes in the blood and the liver was significantly higher in Isoflurane group than Propofol group. However, CHOP in the kidneys was not different between the two groups.

The levels of CHOP from the blood as the baseline status at the induction of anaesthesia had the no significant difference between the two groups [24,014 ± 2,850 (23,437, 21,348-27,836) mean fluorescence intensity (MFI) in the Isoflurane group *vs.* 20,879 ± 1,668 (21,505, 19,150-22,295) MFI in the Propofol group, *p* = 0.077]. After anaesthesia, Isoflurane group had the significant increased level of CHOP [31,499 ± 4,934 (30,733, 26,441-38,087), *p* = 0.039] but Propofol group did not [20,595 ± 1,838 (20,780, 18,866-22,232), *p* = 0.805]. The level of CHOP from the blood after anaesthesia was significantly higher in the Isoflurane group, compared to the Propofol group (*p* = 0.002). The level of CHOP from the liver was significantly higher after anaesthesia in the Isoflurane group [28,342 ± 5,535 (29,421, 233,88-32,756) MFI in the Isoflurane group *vs.* 20,004 ± 2,155 (19,244, 18,197-22,191) MFI in the Propofol group, *p* = 0.020]. However, the level of CHOP from the kidneys after anaesthesia was not different between the two groups (Fig. 1).

The level of ROS from the blood was significantly higher after anaesthesia in the Isoflurane group, compared to the Propofol group [64.68 ± 7.93% (67.50, 56.53-71.44) in the Isoflurane group *vs.* 51.41 ± 7.26% (50.34, 44.57-58.80) in the Propofol group, *p* = 0.025] (Table 1) but no significant differences were observed in the liver or the kidneys (Table 1).

The levels of cytokines from the blood before and after anaesthesia were similar in the two groups (Table 2).

**Figure 2:**
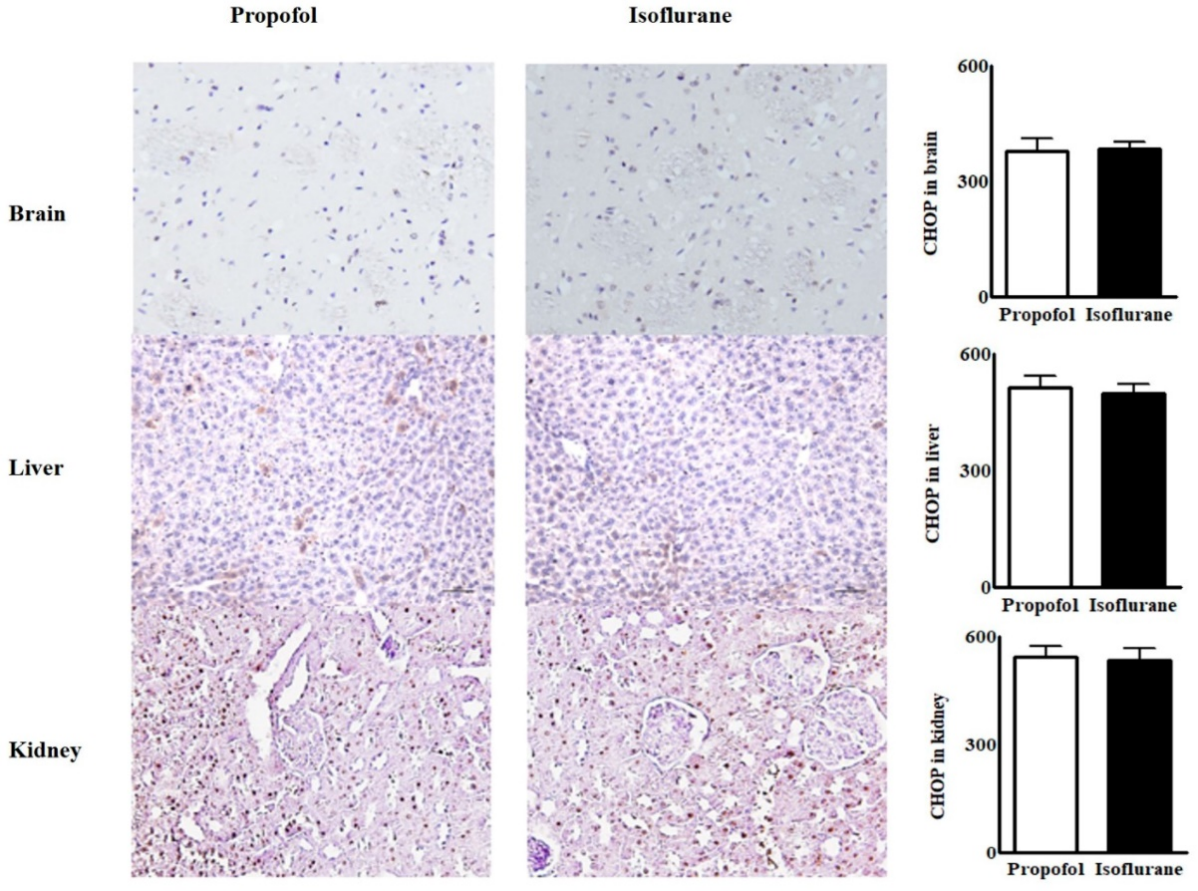
Immunohistochemical assay for endoplasmic reticulum stress marker from the tissues in the brain, the liver and the kidneys. **Abbreviations:** CHOP, CCAAT-enhancer-binding protein homologous protein; MFI, mean fluorescence intensity. CHOP from the tissues in the brain, the liver and the kidneys were similar between the two groups.

Immunohistochemical assays for ER stress marker in tissues from the brain, the liver and the kidneys were similar between the groups (Fig. 2).

**Figure 3:**
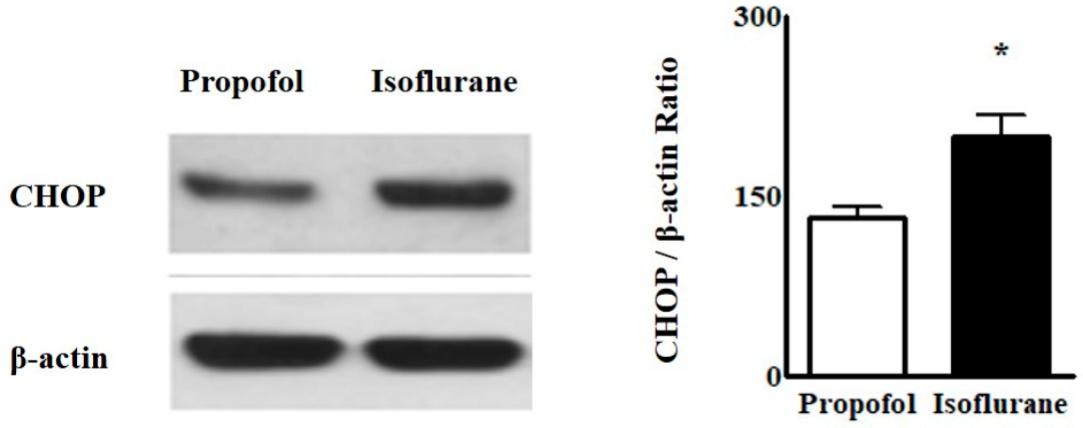
Western blot analyses of endoplasmic reticulum stress from lymphocytes in the blood. **Abbreviations:** CHOP, CCAAT-enhancer-binding protein homologous protein. ^*^p < 0.05. Western blot analyses from lymphocytes in the blood showed the intensity of CHOP was higher in Isoflurane group than in Propofol group.

Western blot analyses for ER stress indicated that the intensity of CHOP from the blood was higher in the Isoflurane group than in the Propofol group [CHOP, 199.8 ± 17.88 (188.50, 192.55-212.70) vs. 131.6 ± 10.01 (145.00, 131.50-125.00), *p* = 0.004] (Fig. 3).

## Discussion

There was a significant higher ER stress from lymphocytes in the blood and the liver of rats under anaesthesia with isoflurane, compared to propofol. They also exhibited a significant higher ROS from lymphocytes in the blood. However, no differences in cytokine levels or immunohistochemical assays in tissues were observed between the two anaesthetic groups.

It is difficult to clarify the pure effects of an anaesthetic agent, separate from surgical stimuli, or the immune response. However, many studies have demonstrated that anaesthetic agents directly or indirectly modulate the immune response 15-18. Although expression of the immune response depends on cell type and host condition, propofol has anti-inflammatory effects 15. It also reduces activation of the hypothalamic-pituitary-adrenal axis and limits increases in cortisol, norepinephrine, and epinephrine, compared to inhalation anaesthetics 16. Consequently, propofol attenuates the surgical stress-induced immune response better than an inhalation anaesthetic agent. Inada* et al*. demonstrated this observation in patients undergoing a craniotomy for clipping of an un-ruptured aneurysm 18. Therefore, we hypothesised that anaesthesia with propofol would be associated with less ER stress than anaesthesia with isoflurane.

ROS in the blood was significantly higher after anaesthesia with isoflurane, compared to anaesthesia with propofol. Huang* et al*. reported that propofol attenuates ROS production during one-lung ventilation in thoracic surgery 19. Seo* et al*. also reported that the expression of ROS is significantly higher after anaesthesia with isoflurane than after propofol during ischemia-reperfusion injury of the myocardium, although the heart injury scores in that study are similar between the two groups 20. In fact, accumulating evidence has demonstrated that propofol inhibits ROS production 21-23. Therefore, our results are reasonable.

Increased ROS are associated with ER stress, which causes mitochondrial dysfunction and increases mitochondrial ROS production. Increased CHOP during ER stress contributes to oxidative stress and the accumulation of ROS 24. Many ER stress-related models have demonstrated that ER stress and oxidative stress, representing ROS production, accentuate each other, interfere with cell function, and activate pro-apoptotic signalling 25-27. Although the level of ROS is not checked, Zhou* et al*. reported that propofol inhibits the activation of protein kinase R like endoplasmic reticulum (PERK)-eukaryotic translation initiation 2A (eIF2A)-activating transcription factor (ATF)-CHOP during ER stress 28. By contrast, isoflurane might induce ER stress by activating caspases and apoptosis 29. Therefore, the increased level of ROS observed under anaesthesia with isoflurane in the present study were associated with increased ER stress, although the mechanism between ER stress and ROS has not be clearly clarified 30-34.

Several conditions in the present study should be considered to clarify the effects of these anaesthetic agents on ER stress. First, no haemodynamic parameters were checked during different planes of anaesthesia. Although the usual dose of anaesthesia was applied, results without similar haemodynamic parameters and similar anaesthetic depth could be different 35-37. Second, several studies have failed to show any meaningful clinical consequences, including histological findings, although differences in the levels of ROS between propofol and isoflurane were checked 20,36-39. Seo* et al*. also reported no differences in the myocardial injury score between propofol and isoflurane during ischemia-reperfusion injury of the myocardium, although a difference in the levels of ROS is verified 20. Third, CHOP was used to check ER stress in the present study. ER stress has several pathways through inositol-requiring protein 1, protein kinase RNA-like ER kinase (PERK), and activating transcription factor (ATF)-6 40. ER stress triggers CHOP, which accumulates through the PERK and ATF-6 pathways. Therefore, determining CHOP activity was sufficient to check the entire pathway for ER stress in the present study, although other markers for ER stress were not evaluated.

In conclusion, ER stress from lymphocytes in the blood and the liver was significantly higher in rats under anaesthesia with isoflurane than propofol. A significant higher ROS from lymphocytes in the blood was observed under anaesthesia with isoflurane than propofol. However, these observations were not associated with any changes of cytokines in the bloods or immunohistochemiscal assays in the tissues.

## Figures and Tables

**Table 1:** Reactive oxygen species (ROS) between Propofol *vs.* Isoflurane groups.

	Propofol group	Isoflurane group	*p-*value
Blood (%)	51.41 ± 7.26 (47.55, 45.40-52.23)	64.68 ± 7.93 (63.37, 59.30-65.58)	0.025
Liver (%)	28.66 ± 4.97 (29.10, 27.44-30.70)	32.14 ± 5.41 (30.57, 28.40-31.74)	0.321
Kidney (%)	14.13 ± 2.20 (15.30, 14.55-15.60)	13.30 ± 3.32 (14.75, 13.84-15.12)	0.654

Data is presented as mean ± standard deviation (median, interquartile range).

**Table 2:** Cytokines in the blood between propofol *vs.* Isoflurane groups.

	Before anesthesia			After anesthesia		
	Propofol	Isoflurane	*p-*value	Propofol	Isoflurane	*p-*value
IL-2 (ng/mL)	100.50 ± 20.51 (108.20, 89.45-113.50)	104.60 ± 30.22 (106.34, 87.56-112.00)	0.807	95.42 ± 22.11 (102.35, 93.40-110.87)	100.60 ± 35.14 (103.11, 84.75-118.20)	0.786
IFN-γ (ng/mL)	210.60 ± 20.98 (205.62, 197.75-215.22)	195.40 ± 16.80 (201.50, 190.30-205.94)	0.242	214.70 ± 29.66 (208.85, 198.60-227.15)	195.30 ± 35.13 (210.90, 190.74-226.45)	0.373
TNF-α (ng/mL)	286.60 ± 21.57 (280.30, 274.52-293.63)	290.90 ± 49.03 (286.80, 265.23-305.64)	0.861	296.70 ± 32.46 (273.90, 289.38-316.20)	299.00 ± 61.32 (270.29, 260.82-302.85)	0.943
TGF-β (ng/mL)	234.40 ± 21.19 (240.12, 231.50-246.70)	248.50 ± 17.11 (246.74, 238.19-252.20)	0.282	248.30 ± 19.07 (237.05, 230.35-249.36)	252.20 ± 22.62 (240.50, 236.10-254.77)	0.775

Data is presented as mean ± standard deviation (median, interquartile range). **Abbreviations:** IL, interleukin, IFN, interferon; TNF, tumor necrosis factor; TGF, transforming growth factor.
